# Ecological Engineering of the Human Gut Microbiome: A Narrative Review and Framework for Next-Generation Therapeutics

**DOI:** 10.3390/microorganisms14092001

**Published:** 2026-09-09

**Authors:** Antonio Díaz, Gissel García, Raúl De Jesús Cano

**Affiliations:** 1 Statistical and Research Department, Hermanos Ameijeiras Clinical and Surgical Hospital, Calle San Lázaro No. 701, Esq. a Belascoaín, Centro Habana, La Habana 10400, Cuba; adm12101972@gmail.com; 2 Pathology Department, Hermanos Ameijeiras Clinical and Surgical Hospital, Calle San Lázaro No. 701, Esq. a Belascoaín, Centro Habana, La Habana 10400, Cuba; gisselgarcia2805@gmail.com; 3 Biological Sciences Department, California Polytechnic State University, San Luis Obispo, CA 93407, USA; 4 Academia de Ciencias de Cuba, La Habana 12400, Cuba

**Keywords:** gut microbiome, ecological succession, spore-forming probiotics, synbiotics, and community assembly

## Abstract

The human gut microbiome is a complex adaptive ecosystem whose functions arise from interactions among microbial populations rather than from isolated taxa. Nevertheless, many microbiome-directed interventions still rely on administering individual strains, with limited consideration of the ecological processes governing community assembly, succession, and resilience. This review integrates evidence from microbial ecology, comparative genomics, systems biology, mechanistic physiology, and clinical microbiome research to propose a testable framework for ecologically engineering the human gut microbiome. Within this framework, selected spore-forming probiotics are hypothesized to function as transient pioneer organisms that modify intestinal physicochemical and metabolic conditions, thus facilitating the establishment and activity of functionally complementary microbial populations delivered through rationally designed synbiotic consortia. The proposed process comprises five stages: pioneer activity, niche remodeling, facilitated community assembly, functional-network stabilization, and the emergence of host-associated outcomes. Available genomic, physiological, and clinical observations support the biological plausibility of individual components of this model but do not yet demonstrate directed ecological succession as a complete causal process. Accordingly, the framework distinguishes established evidence from ecological inference and generates experimentally testable predictions of temporal niche modification, metabolic cross-feeding, functional redundancy, resilience after treatment withdrawal, and host metabolic responses. This ecological perspective shifts the objective of microbiome therapeutics from transient strain supplementation toward the predictable modulation of community trajectories, providing an experimental foundation for developing more resilient, mechanism-based interventions.

## 1. Introduction

The human gut microbiome is a complex microbial ecosystem that contributes to host metabolism, immune regulation, epithelial homeostasis, and resistance to pathogen colonization [1,2,3,4,5,6,7,8]. Individual microorganisms do not produce these functions independently. They arise from interactions among microbial populations and from the combined effects of their genes, metabolic products, and responses to the intestinal environment [9,10]. As such, identifying the microorganisms present in a sample cannot fully explain the microbiome’s biological activity. It is also necessary to determine what these organisms are doing, how they interact, and how those interactions change after a disturbance or therapeutic intervention [11,12,13].

This ecological view of the microbiome has important consequences for designing probiotics and other microbiome-directed therapies. Many probiotic organisms have favorable safety profiles, and some have produced measurable clinical benefits [14,15,16,17,18,19,20,21,22,23,24].

However, their effects vary considerably among individuals, and administered organisms are often detected only for a limited period. The absence of permanent colonization does not necessarily indicate therapeutic failure [25,26]. A microorganism that passes through the intestine may still produce metabolites, compete for nutrients, alter local physicochemical conditions, interact with resident organisms, or influence host signaling [27,28]. The more relevant question, therefore, is whether an administered microorganism produces an ecological effect while it remains active.

The resident microbiome presents a considerable barrier to such an effect [29,30]. The adult intestine contains a dense and established microbial community in which organisms compete for nutrients and attachment sites, produce inhibitory compounds, and modify the environment in ways that favor some populations while excluding others [31,32,33,34,35,36]. Diet, medications, host physiology, inflammation, and the initial microbiome composition further influence the response to an intervention. The order in which organisms and microbial functions become established may also be important. These priority effects help explain why the same formulation may produce different microbiological or clinical outcomes in different recipients. They also indicate that adding microorganisms without considering the recipient community’s ecological state may not be sufficient to produce a predictable response [37].

Efforts to address these limitations are increasing through multi-strain probiotics, synbiotics, defined microbial consortia, live biotherapeutic products, engineered microorganisms, targeted bacteriophages, and fecal microbiota transplantation [38,39]. These approaches demonstrate that the microbiome can be modified at several biological levels. Much of the evidence, however, still relies on changes in the relative abundance of individual taxa [40,41,42]. Such measurements are useful, but they do not necessarily reveal the ecological process responsible for the observed change. An increase in a bacterial population may result from direct growth, reduced competition, altered substrate availability, a change in host physiology, or normal temporal variation [30,43,44]. Likewise, detecting an administered strain does not establish that it has become functionally integrated into the resident community.

Ecological succession may provide a useful framework for examining these processes. In natural ecosystems, early organisms can modify their environment and thereby affect the establishment or activity of later-arriving organisms [45,46,47,48,49,50,51]. This process may involve facilitation, inhibition, or tolerance, but it does not always produce a more diverse or stable community, nor does it necessarily move the ecosystem toward a desirable state. The intestinal microbiome also differs from an unoccupied habitat because it is already densely populated and is continuously influenced by diet and the host [52,53]. Nevertheless, an administered microorganism could perform a pioneer-like function if its activity alters local conditions and if those changes precede and contribute to the response of other microbial populations.

Spore-forming probiotics are plausible candidates for such a role. Their endospores can survive exposure to gastric acidity and bile and may subsequently germinate in the intestine [54,55]. Following germination, their vegetative cells may affect nutrient availability, pH, redox conditions, oxygen concentration, antimicrobial activity, or metabolite production [56,57]. These changes could influence resident microorganisms or organisms delivered later as part of a complementary consortium. Permanent engraftment would not be required. The ecological contribution of the spore-forming organism could be transient, provided that its activity changes the conditions under which other microbial functions are expressed.

At present, no single study has shown that a spore-forming probiotic can initiate and direct the complete succession process proposed here. Genomic and physiological studies indicate that these organisms possess traits that could modify the intestinal environment [58]. Clinical investigations of probiotics and synbiotics have also reported changes in metabolic markers and microbiome composition [59,60]. These findings are important, but each represents only part of the proposed process. Demonstrating succession will require evidence that the spore-forming organism becomes active first, produces a measurable change in the intestinal environment, and that this change influences the organisms or microbial functions that follow.

The purpose of this review is to determine whether these separate lines of evidence can be brought together into a useful ecological model. We propose examining the process in five stages: pioneer activity, niche remodeling, facilitated community assembly, stabilization of microbial functions, and effects on the host. We refer to the deliberate use of these activities and interactions as ecological engineering. This does not imply that every intervention will produce succession or that a stable microbiome will always be beneficial. Some interventions may have no ecological effect, while others could move the community in an undesirable direction. The model is useful only if it distinguishes these outcomes from transient passage of the administered organisms, independent strain effects, dietary responses, and normal microbiome variation. If the proposed sequence can be demonstrated, microbiome therapeutics could be designed around complementary ecological functions rather than relying primarily on individual-strain properties.

## 2. Review Scope and Conceptual Approach

This review identified the scientific literature through searches of PubMed and Google Scholar, last updated on 28 August 2026. We used search terms individually and in combination, employing Boolean operators to combine controlled vocabulary and free-text terms. Example queries included:

• “gut microbiome” AND “ecological succession”
• “community assembly” AND “microbiome”
• “ecosystem engineering” AND “probiotics”
• “spore-forming probiotics” OR “synbiotics”
• “microbiome resilience” OR “colonization resistance”
• “priority effects” AND “intestinal microbiota”
• “live biotherapeutic products” AND “clinical trial”
• “metabolic cross-feeding” AND “microbiome”
• “host–microbiome interactions”

The search aimed to identify evidence relevant to the ecological processes that may govern the gut microbiome’s response to therapeutic intervention. Searches covered publications from 2000 to 2026, including earlier foundational ecological studies that established principles or methods central to the proposed model.

The literature reviewed included experimental studies, clinical investigations, genomic and physiological studies, ecological models, consensus documents, and previous reviews. Foundational studies that defined ecological principles or experimental methods central to the proposed model, and more recent publications were included to assess how these principles apply to probiotics, synbiotics, and other microbiome-directed therapies.

Studies were included if they:

• Reported experimental, clinical, genomic, or ecological data relevant to microbiome processes;
• Provided mechanistic or conceptual insight into succession, redundancy, resilience, or host-associated outcomes; and
• Were peer-reviewed articles, consensus documents, or authoritative reviews.
• We excluded studies that were not peer-reviewed, were conference abstracts without full data, or lacked relevance to ecological processes.

This is a narrative and conceptual review, not a systematic review or meta-analysis; we did not quantitatively pool clinical outcomes. Studies were selected for their relevance to one or more elements of the proposed framework, including survival and germination of spore-forming organisms, modification of the intestinal environment, metabolic cooperation, community assembly, functional redundancy, resilience, and host-associated effects.

This literature was used to examine whether observations from different areas of microbiome research could support a common ecological model, developed by identifying where these separate lines of evidence agree, where they remain incomplete, and what experiments would be needed to distinguish the proposed process from alternative explanations. Important gaps remain, particularly in establishing the order and causality of the proposed events. Accordingly, the framework presented here should be regarded as a model intended to guide future experiments rather than as an established mechanism of microbiome therapy.

## 3. Current Strategies for Microbiome Engineering: Progress and Ecological Limitations

### 3.1. From Individual Strains to Microbial Communities

Probiotic development has traditionally focused on selecting individual strains with desirable properties [61,62,63]. These properties may include survival during gastrointestinal transit, adhesion to intestinal surfaces, production of antimicrobial compounds, modulation of immune responses, or the ability to perform a particular metabolic function [36,38,39,46]. This approach has produced probiotics with established safety records and documented benefits in selected clinical conditions. It has also provided much of the physiological and genomic information that underpins more complex formulations.

The activity of a probiotic strain, however, cannot be separated from the microbial community into which it is introduced [64,65]. An organism that performs well under laboratory conditions may behave differently in an intestine where substrates are limited, attachment sites are occupied, and resident microorganisms compete for the same resources. The response may also differ according to diet, medication use, intestinal transit time, host physiology, and the initial composition of the microbiome [66]. These factors do not make the strain-centered approach invalid, but they limit how much a strain’s properties alone can predict its effect in a microbial community.

Long-term persistence has often been considered a desirable characteristic of a probiotic [67,68]. Persistence may be beneficial in some circumstances, but permanent colonization is not required for every therapeutic effect [30,69]. A transient organism may remain metabolically active long enough to influence resident microorganisms or the host [70,71]. The more important consideration is whether that activity is reproducible and whether it produces the intended biological effect. This distinction shifts attention from the presence of the administered organism to the changes that occur while it is present.

### 3.2. Synbiotics and Rational Microbial Consortia

The development of prebiotics and synbiotics expanded probiotic therapy by combining microorganisms with substrates intended to support microbial activity [72,73,74]. Early synbiotic formulations often paired established probiotic strains with broadly fermentable carbohydrates. Such combinations may be useful, but the presence of a microorganism and a fermentable substrate does not, by itself, demonstrate a cooperative ecological relationship [75].

A rationally designed synbiotic should be based on a defined functional connection between its components. A substrate may support the growth or metabolic activity of an administered organism, or it may be converted into products used by resident microorganisms. Likewise, one member of a consortium may transform a nutrient into an intermediate another member requires. These cross-feeding relationships can distribute metabolic functions among several populations and may allow the consortium to perform activities that no member can perform alone.

Defined microbial consortia extend this principle by selecting organisms for complementary functions rather than simply increasing the number of strains in a formulation [60]. The objective is not necessarily to reproduce the taxonomic complexity of a healthy microbiome. It is to provide a limited set of organisms capable of initiating or supporting functions that are absent, reduced, or poorly expressed in the recipient community. This approach requires knowledge of substrate use, metabolite production, environmental requirements, and possible competition among the selected organisms.

A consortium’s behavior depends on the resident microbiome [6,76]. Organisms that cooperate in vitro may compete in vivo, while a metabolic product that supports one population may inhibit another. Resident taxa may also supply functions, making an administered member redundant. For these reasons, consortium design should include not only the characteristics of its members but also the ecological conditions under which their proposed interactions are expected to occur.

### 3.3. Live Biotherapeutic Products and Engineered Microorganisms

Live biotherapeutic products have introduced greater precision into the development and regulatory evaluation of microbial therapies [77,78]. They are generally composed of defined organisms manufactured under controlled conditions to prevent, treat, or manage disease. Their development requires careful attention to identity, purity, potency, stability, antimicrobial resistance, and safety. These requirements represent an important advance over products with poorly characterized composition or biological activity.

Many live biotherapeutic products are still developed around the activity of one organism or one principal mechanism [79,80]. Others use defined mixtures intended to replace missing functions or suppress undesirable populations [81,82]. Genetically engineered microorganisms provide an additional level of control by introducing specific sensing, metabolic, or delivery functions [83,84]. These organisms may be designed to detect local signals, consume selected substrates, produce therapeutic molecules, or interfere with pathogenic processes.

Precision at the level of the engineered organism does not eliminate the ecological problem [85]. Product activity may still depend on nutrient availability, spatial access, competition, host responses, and interactions with the resident microbiome. An engineered function that is readily detected in culture may be reduced or lost in the intestine. Ecological measurements should therefore accompany measurements of strain survival and product expression [86].

### 3.4. Fecal Microbiota Transplantation

Fecal microbiota transplantation (FMT) provides the clearest clinical example of a therapeutic effect produced by transferring a microbial community rather than an individual strain [86,87]. Its effectiveness in recurrent *Clostridioides difficile* infection shows that large-scale modification of the intestinal microbiome can restore colonization resistance and alter disease outcomes [88]. FMT also shows that microbial functions can be transferred even when the individual organisms responsible for those functions are not fully defined [89].

FMT is not, however, a controlled form of ecological engineering. Donor material contains a complex and incompletely characterized mixture of microorganisms, genes, metabolites, bacteriophages, and other biological components. The contribution of each component is difficult to determine, and the resulting community may vary by donor and recipient. Safety screening reduces identifiable risks but does not fully control the biological material being transferred [90].

The success of FMT establishes that ecosystem-level intervention is possible. It does not show that a small consortium can reproduce the same effect, nor does it prove that directed succession drives the clinical outcome [91,92]. The challenge is to determine whether defined organisms and substrates can reproduce the relevant functions of a com [93,94] plex community, with activities that can be measured, controlled, and manufactured consistently.

### 3.5. The Remaining Ecological Gap

Current microbiome therapies span a wide range, from individual probiotics and dietary substrates to engineered organisms, defined consortia, and whole-community transfer [38,39,72,73,74,77,78,86,87,95,96,97]. Each approach alters some component of the intestinal ecosystem [36,38,39,45,72,73,74,97,98]. The sequence of events linking the administered intervention to the resulting microbial and host response remains poorly defined [99,100].

A change in community composition may indicate that an intervention had an effect, but it does not explain how the change occurred [40,42]. The administered organisms may act directly on the host, compete with resident organisms, alter nutrient availability, produce inhibitory compounds, or change environmental conditions [27,28,31,32,34,35,36,101,102]. Several of these processes may occur simultaneously [9,10]. Without temporal measurements and appropriate controls, it is difficult to determine which event initiated the response and which changes occurred secondarily [99,100].

We use the term ecological engineering to describe the deliberate use of these processes to influence the direction of microbial-community change [34,35]. Under this definition, the objective is not simply to add beneficial microorganisms or remove undesirable ones. Instead, it aims to alter the conditions and interactions that determine which microbial functions can become established and maintained [34,35,103]. The following section examines how such a process could occur through a sequence beginning with pioneer activity and ending, under favorable conditions, with a measurable effect on the host.

The principal strategies used to modify the gut microbiome differ in their composition and immediate objectives, but each encounters limitations imposed by the recipient ecosystem. Table 1 summarizes these differences and their implications for the proposed framework.

## 4. The Five-Stage Ecological Engineering Framework

**Figure 1:**
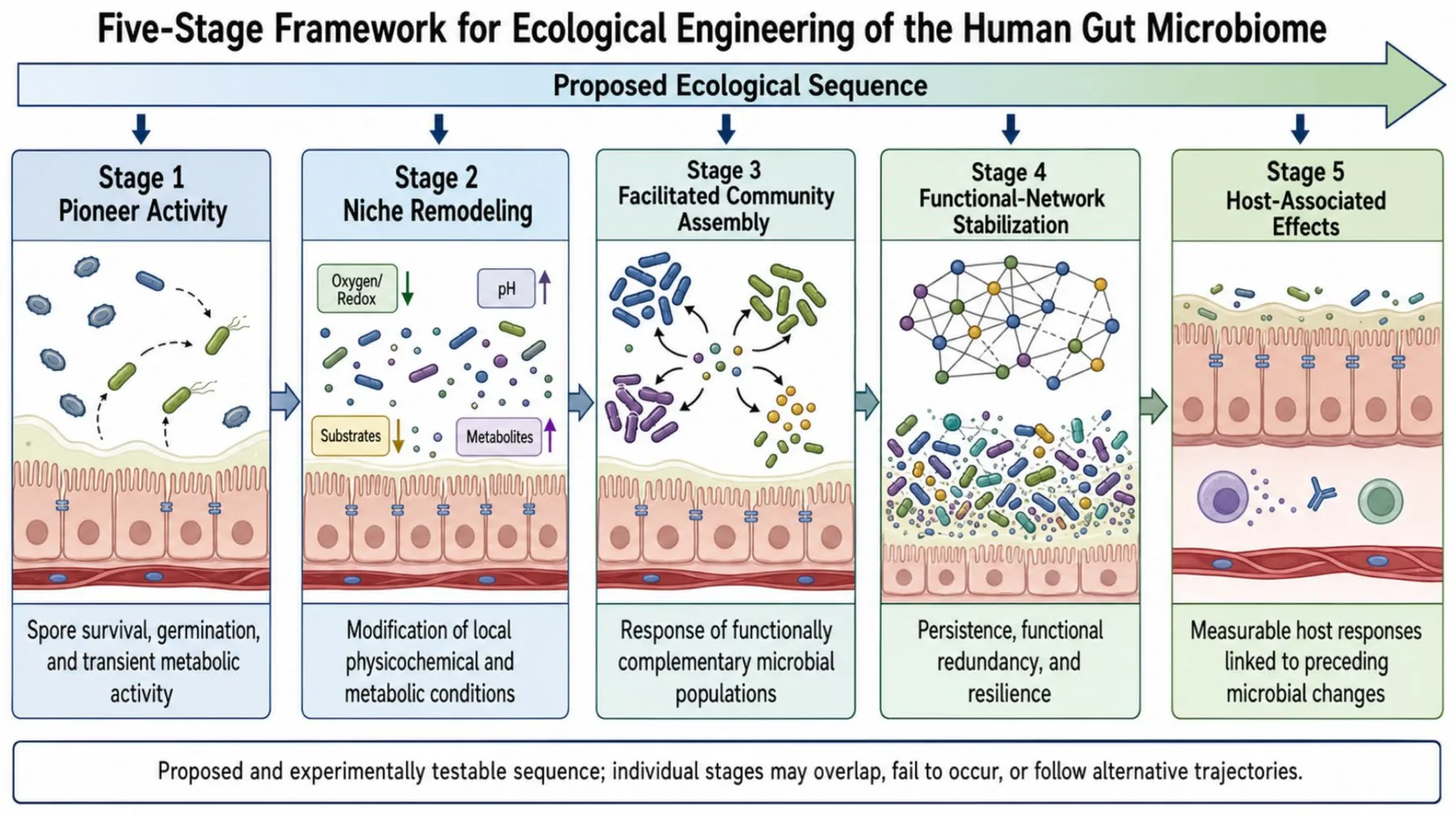
Five-stage framework for ecological engineering of the human gut microbiome. The proposed sequence begins with the germination and metabolic activity of transient pioneer organisms, followed by modification of the intestinal environment, facilitated assembly of functionally complementary microbial populations, stabilization of microbial functions, and measurable effects on the host. The stages represent a proposed sequence for experimental evaluation and should not be interpreted as a process that has already been demonstrated in its entirety. Progression through the framework may vary with the baseline microbiome, diet, host physiology, medications, intervention composition, and other ecological conditions. The large horizontal arrow and arrows between panels indicate the proposed progression through the five stages. Upward and downward arrows in Stage 2 indicate increases and decreases, respectively, in the indicated physicochemical or metabolic variables. Curved and dashed arrows illustrate microbial activation, movement, or recruitment, while connecting lines in Stage 4 represent functional interactions within the microbial network. The blue-to-green color transition distinguishes successive stages of the proposed ecological sequence. The different colors assigned to microorganisms and network nodes are schematic and distinguish microbial populations or functional groups; they do not represent specific taxa unless otherwise indicated. This figure was generated with the assistance of a generative artificial intelligence tool based on the authors’ specifications and was reviewed and edited by the authors, who take full responsibility for its content.

The proposed framework consists of five related stages: pioneer activity, niche remodeling, facilitated community assembly, functional-network stabilization, and host-associated effects (Figure 1). These stages are presented sequentially to define the events required for directed ecological succession. In practice, they may overlap, fail to occur, or follow different trajectories depending on initial microbiome structure, intervention composition, diet, medication use, and host physiology. The model therefore describes a hypothesis to be tested rather than an inevitable response to treatment.

### 4.1. Stage 1: Pioneer Activity

The first stage begins when administered spore-forming bacteria survive passage through the upper gastrointestinal tract, germinate, and become metabolically active. Their proposed role is not necessarily to establish permanent populations. Rather, they may act transiently during a period when their metabolic activities can alter the conditions other members of the community encounter.

Endospore formation provides an obvious advantage during gastrointestinal transit [109,110]. The dormant endospore resists acid, bile, desiccation, and other environmental stresses that would reduce the viability of many vegetative bacteria. Strain-specific studies of *Alkalihalobacillus clausii*, *Heyndrickxia coagulans*, and others have reported survival under simulated gastrointestinal conditions and genomic characteristics consistent with their use as orally administered microorganisms [93,94,111]. These observations support survival and potential metabolic activity after ingestion. They do not, however, establish where germination occurs in vivo, how long the resulting vegetative populations remain active, or whether their activity precedes changes in the resident microbiome.

Several genera of spore-forming bacteria are commonly used as probiotics. The genus *Bacillus* remains the most widely recognized, with species such as *Bacillus subtilis*, *Bacillus licheniformis*, and *Bacillus indicus* having been extensively studied for their safety and physiological effects [60,61,104,105]. In recent taxonomic revisions, some former *Bacillus* species have been reclassified into new genera: *Heyndrickxia coagulans* (formerly *Bacillus coagulans*) and *Alkalihalobacillus clausii* (formerly *Bacillus clausii*) are prominent examples of spore-forming probiotics with documented safety and functional properties [98,99,100]. These organisms share the common feature of producing durable endospores that resist gastric acid and bile, but they differ in their metabolic capabilities, oxygen tolerance, and the specific bioactive compounds they produce. For example, *H. coagulans* has been reported to produce lactic acid and to modulate gastrointestinal transit, while *A. clausii* produces antimicrobial peptides and has been associated with immune-modulating effects [57,93,94,111]. These strain-specific differences determine their potential ecological roles as pioneer organisms.

The central prediction of this stage is therefore temporal. Spore germination and vegetative activity should occur before the environmental and community changes assigned to the subsequent stages. This prediction can be examined by combining strain-specific quantification with measurements of germination, transcriptional activity, and metabolite production at closely spaced intervals after administration. Detection of spores or strain DNA in feces alone would not be sufficient, because neither establishes germination nor metabolic activity within the intestine.

### 4.2. Stage 2: Niche Remodeling

Following endospore germination, the vegetative cells become metabolically active and begin to alter their immediate surroundings [112]. They consume available substrates, produce organic acids and other metabolites, and may inhibit competing organisms. These activities may change the conditions under which neighboring microbial populations must function. This underpins the proposed niche-remodeling stage.

Oxygen availability is particularly important in the intestine [102,113]. The healthy colon is predominantly anaerobic, but inflammation and impaired epithelial metabolism can increase oxygen availability and other respiratory electron acceptors near the mucosal surface. These conditions favor facultative anaerobes, including members of the Enterobacteriaceae, and may restrict obligate anaerobes associated with normal fermentation and short-chain-fatty-acid production [114,115]. Oxygen consumption by metabolically active pioneer organisms could help restore a lower redox potential. Organic-acid production and bacteriocin activity could provide additional selective pressures [116,117].

Specific examples illustrate how these activities may occur at the strain level. *A. clausii* AO1125 produces antimicrobial peptides that inhibit the growth of competing facultative anaerobes in vitro, an activity consistent with a role in reducing pathobiont abundance in the intestinal lumen [93,111]. *H. coagulans* AO1167B consumes oxygen during its vegetative metabolism, which may help lower local redox potential and create conditions more favorable to obligate anaerobes [94]. Additionally, *H. coagulans* has been shown to ferment carbohydrates to lactic acid and other organic acids, potentially lowering local pH and inhibiting acid-sensitive enteropathogens [94,118,119]. These strain-specific properties are supported by in vitro assays and genomic annotations, although their occurrence and significance in vivo remain to be confirmed in human studies.

Evidence suggests that individual strains can carry out some of these activities under laboratory conditions [120,121]. Whether they do so within the intestine, and whether the resulting changes are sufficient to influence other members of the microbiome, remains uncertain. Genomic analysis can identify a strain’s metabolic capabilities, but the presence of relevant genes does not tell us when, where, or to what extent they are expressed after ingestion.

This stage predicts a measurable environmental change following pioneer activity. Such a change might include reduced oxygen availability, altered redox potential, localized pH changes, depletion of particular substrates, production of organic acids, or inhibition of competing organisms. It must also precede the response of the organisms presumed to benefit from niche remodeling. Without this temporal relationship, subsequent changes in community composition cannot confidently be attributed to pioneer activity.

### 4.3. Stage 3: Facilitated Community Assembly

If pioneer activity produces favorable environmental changes, microbial populations introduced with a synbiotic consortium—or already present at low abundance in the recipient—may become more metabolically active or increase in abundance. This constitutes facilitation only if these populations respond to conditions created by the earlier organisms. Their simultaneous detection is not, by itself, evidence of an ecological relationship.

The proposed process may involve several forms of facilitation. Oxygen consumption may favor strict anaerobes [102]. Organic acids or other fermentation products may serve as substrates for secondary consumers [122]. Antimicrobial compounds may reduce competition from susceptible organisms. Prebiotic components may provide selectively utilized substrates, and the metabolic products of one population may support the growth or activity of another. These interactions could enable community functions that strains acting independently would not achieve [123,124].

For instance, mannitol produced by certain spore-forming strains via endogenous glucose conversion [125] could serve as a fermentable substrate for butyrate-producing anaerobes such as *Faecalibacterium prausnitzii* or *Roseburia* species, illustrating a potential cross-feeding interaction between pioneer spore-formers and secondary community members [126,127,128]. Similarly, lactate generated by *H. coagulans* during carbohydrate fermentation may be used by lactate-consuming butyrate producers, a metabolic cross-feeding pathway that has been characterized in related gut microbiota [128].

Clinical studies of multicomponent probiotic and synbiotic formulations have reported changes in microbiome composition and metabolic biomarkers. These findings are compatible with community-level effects, but they do not yet show that pioneer-mediated facilitation produced the observed changes. Demonstrating facilitation will require comparisons among the pioneer organisms alone, the secondary consortium alone, the complete formulation, and appropriate controls. A true facilitative interaction should produce an effect in the combined intervention that cannot be explained by adding the independent effects of its components.

Several ecological processes could contribute to secondary community assembly. They include modification of the local environment, exchange of metabolic products, use of complementary substrates, and relief from competition. Table 2 summarizes these proposed interactions, the functions they may support, and the observations needed to determine whether they occur during treatment.

The principal prediction of Stage 3 is that community assembly will depend upon both formulation and order of exposure. If niche remodeling is necessary, administering the pioneer organisms before or with the secondary consortium should produce a different outcome from administering the consortium alone. Reversing the order, altering the interval between components, or disabling a proposed pioneer function should weaken or eliminate the effect.

### 4.4. Stage 4: Functional-Network Stabilization

The appearance of a newly assembled community does not mean that it will persist. Administered organisms may simply pass through the intestine, and changes among resident populations may disappear after treatment ends. Stage 4 addresses whether the functions and interactions that emerged during the earlier stages are maintained.

Stabilization is not assumed to be either automatic or necessarily beneficial: a community can also stabilize into a persistently altered but non-beneficial or dysbiosis-adjacent configuration, and stabilizing a function does not, by itself, mean that the resulting network is resilient in a favorable direction. Distinguishing beneficial from non-beneficial stabilization requires the same functional and compositional monitoring described below, interpreted against pre-treatment baseline and clinical outcome data rather than assumed from persistence alone. This caution is not merely theoretical: several randomized probiotic trials have failed to detect lasting, significant alterations in gut microbiota composition after treatment ends, underscoring that apparent Stage 4 stabilization must be confirmed empirically rather than assumed [136].

Functional stability may develop when multiple microbial populations distribute important metabolic activities. This functional redundancy can allow a community to maintain its output despite changes in individual-taxa abundance [137,138]. Cross-feeding may provide a second source of stability. Lactate and acetate produced by bifidobacteria and lactic-acid bacteria, for example, can be used by obligate anaerobes that produce butyrate, including members of *Faecalibacterium*, *Roseburia*, *Anaerobutyricum*, and related genera [126,127,128].

The published Phase I safety trial of *H. coagulans* AO1167B provides direct evidence of safety and gastrointestinal resilience, but it did not include detailed microbiome profiling, intestinal viability assessments, or host biomarkers such as *Faecalibacterium,* Homeostatic Model Assessment of Insulin Resistance (HOMA-IR), or lipopolysaccharides (LPS). Any ecological or metabolic associations therefore remain hypothetical and require targeted, strain-resolved, longitudinal studies to validate.

Stabilization does not require every individual to develop the same microbial composition. Different organisms can perform many of the same metabolic functions, and the organisms responsible for those functions may vary from one person to another. For this reason, changes in taxonomic abundance provide only part of the evidence. It is also necessary to determine whether important metabolic activities persist and whether the community retains them after disturbance.

The strongest evidence for Stage 4 would be maintaining an altered functional state after treatment withdrawal or following a defined perturbation. If the observed changes disappear immediately when administration stops, the intervention may have produced a temporary pharmacological or nutritional effect. However, stability alone does not guarantee benefit. A resilient network may preserve undesirable activities, and redundancy may protect maladaptive functions as well as beneficial ones. For this reason, Stage 4 requires longitudinal validation to confirm that stabilization represents a favorable ecological outcome, rather than persistence of dysbiosis.

#### Baseline Microbiome and Priority Effects

Whether the proposed sequence continues beyond its early stages will depend in part on the microbiome already present. Resident organisms can either support newly introduced organisms or prevent them from becoming established. Ecologists call this influence of earlier inhabitants on later arrivals a priority effect. Such effects may account for some of the differences observed when the same microbiome intervention is given to different individuals.

A microbiome depleted by antibiotics may offer limited colonization resistance but may also lack organisms required for cross-feeding [139]. A diverse microbiome dominated by facultative anaerobes may contain the necessary functional capacity but resist displacement or environmental change. In a comparatively stable microbiome, the intervention may produce little taxonomic change while still altering the activity of existing populations [95]. These conditions should not be treated as equivalent starting points.

We predict that baseline community structure and function will explain a meaningful proportion of treatment variability. Baseline measurements may eventually allow selection of intervention components, sequence, or dose according to the recipient’s ecological state. At present, however, this remains a research objective rather than a clinically validated basis for personalization.

### 4.5. Stage 5: Host-Associated Effects

In the final stage, changes in the microbial community composition begin to affect the host. The response may be detected in the intestinal barrier, in inflammatory activity, or in the metabolic regulation of glucose and lipid metabolism. Evidence must connect these responses to the microbial activities described in the preceding stages. Without this evidence, they are simply clinical observations whose relationship to the proposed ecological sequence is uncertain.

Short-chain fatty acids provide one possible link. Butyrate serves as an energy source for colonocytes and supports epithelial metabolism and the maintenance of a low-oxygen mucosal environment. Acetate and propionate participate in additional metabolic and signaling pathways, including those involving free-fatty-acid receptors [15,16]. Changes in microbial metabolites may also affect tight-junction integrity, immune activity, and exposure to microbial products such as lipopolysaccharide.

Clinical studies of EDC-HHA01 and other formulations have reported changes in HOMA-IR, circulating lipopolysaccharide, and related metabolic measurements [60,140,141,142]. These findings are encouraging and consistent with parts of the model proposed here. However, they do not indicate how the changes occurred. In particular, the studies were not designed to track ecological events from spore germination through modification of the intestinal environment and, eventually, a host response.

Establishing that sequence will require measurements taken at several points during and after treatment. First, researchers will need to determine when pioneer organisms become active and whether their activity alters the surrounding environment. Researchers can then track changes in other microbial populations and their metabolic products, as well as the host response. The proposed mechanism would be more convincing if preventing one early event also prevented the expected downstream events. Of the five stages, the link between microbial activity and host response will probably be the hardest to establish.

While host immune and metabolic responses may begin during earlier stages, the distinction between Stage 4 and Stage 5 lies in the required level of evidence. Stage 4 is defined by the persistence and resilience of microbial functions within the community, independent of host outcomes. Stage 5 begins when those microbial functions can be experimentally connected to measurable host responses. Demonstrating this connection will require longitudinal sampling at multiple time points, linking microbial activity to subsequent host physiology, and showing that disrupting earlier microbial events alters the host outcome.

In practice, researchers can test this boundary directly. A Stage 4 endpoint would be confirmed by measuring the targeted microbial function—for example, butyrate production, cross-feeding intermediates, or facultative-anaerobe abundance—at the community level before, during, and after a defined perturbation such as treatment withdrawal or an antibiotic challenge, independent of any host measurement; persistence or rapid recovery of the function following the perturbation would satisfy the Stage 4 criterion. A Stage 5 endpoint requires the same longitudinal design, extended to paired host measurements such as HOMA-IR, circulating LPS, or intestinal permeability markers collected at matched time points, along with a demonstrated temporal and statistical association between the microbial and host trajectories. Critically, an intervention that alters host outcomes without a corresponding, temporally prior change in the targeted microbial function would indicate that the effect did not proceed through the proposed ecological pathway, allowing Stage 4 and Stage 5 to be dissociated experimentally even when both processes begin close together in time.

### 4.6. Factors That May Alter the Ecological Trajectory

The proposed sequence should not be expected to occur in every individual. Spores may pass through the intestine without germinating in sufficient numbers, or vegetative cells may become active without appreciably altering their surroundings. Even when the intestinal environment is altered, the resident community may not respond as predicted. Any changes that do occur may disappear after treatment is discontinued, and persistence of a microbial change does not necessarily mean it will benefit the host.

These differences likely depend on the intestine’s condition at the start of treatment. Diet and medication use will affect the substrates available to the community, while intestinal transit, inflammation, and the resident microbiome’s composition will affect the survival and activity of the administered organisms [143,144,145]. As a result, a formulation that promotes the desired response in one person may have little effect in another. It may even shift the microbiome along a different trajectory. These possibilities are not exceptions to be dismissed. They are outcomes that must be included when testing the model, particularly if microbiome changes are to be attributed to ecological engineering rather than normal variation within the intestinal community.

### 4.7. Experimental Evaluation of the Framework

A decisive evaluation of the framework requires more than comparing microbiome profiles before and after treatment. The sequence of events must be established, and the dependence of each stage on the preceding one must be tested. This will require longitudinal sampling, strain-resolved analysis, measurements of microbial gene expression and metabolites, assessment of physicochemical conditions within the intestine, and controlled comparisons of the intervention’s individual and combined components.

To strengthen the distinction between different types of support, we now classify the available evidence according to the following hierarchy:

1. Direct evidence: observations pertaining to the specific strains or formulations under discussion.
2. Indirect evidence: findings from related organisms or interventions that suggest mechanistic plausibility.
3. General ecological precedent: broader principles from microbial ecology that provide conceptual context.
4. Pure hypothesis: speculative links that remain to be experimentally tested.
5. This categorization clarifies that while individual components of the framework are supported by direct or indirect findings, the causal sequence as a whole remains hypothetical until longitudinal, mechanistic studies validate it. Table 3 summarizes the principal predictions for each stage, along with the experimental approaches needed to test them.

## 5. Discussion

### 5.1. From Probiotic Administration to Ecological Intervention

When a microorganism enters the intestine, it encounters an already established microbial community. Whether it survives and becomes active depends on that community, as well as on host physiology, available nutrients, and prior exposures. The introduced organism may also alter the conditions it encounters. Therefore, probiotic activity cannot be explained entirely by studying the organism in isolation. It must also be examined in the context of the intestinal ecosystem.

This difference has practical importance. Probiotic strains have commonly been selected based on characteristics measured in pure culture, such as acid and bile tolerance, adhesion, antimicrobial activity, and metabolite production [146,147,148]. These characteristics tell us something about the organism, but not necessarily what it will do once it enters an established microbial community. A strain that performs well in the laboratory may have little effect in the intestine if the required substrates are unavailable or resident organisms restrict its activity. Its metabolic products may also matter little unless other members of the community can use them.

The framework proposed here examines what happens after the strain is administered. For a pioneer organism, the key question is whether its activity alters the conditions that other microorganisms encounter. The same reasoning applies to a synbiotic consortium. Its usefulness depends less on the number of components it contains than on whether those components can function together under intestinal conditions.

This interpretation does not make strain identity unimportant. Strain identity determines metabolic capacity, stress tolerance, safety, and the range of possible interactions. The difference is that these properties are evaluated in relation to an ecological role. A strain intended to serve as a pioneer should be selected for its ability to become active under the relevant intestinal conditions and to produce a measurable change in the surrounding environment. A strain intended to participate later in the sequence should be able to use the substrates or conditions created during earlier stages.

### 5.2. What the Available Evidence Establishes

The framework draws on observations from several areas of microbiome research. Selected strains have been shown to survive gastrointestinal transit and then return to vegetative growth. Once active, these organisms enter an intestinal environment responsive to inflammation, epithelial metabolism, diet, and microbial activity [114,115]. They also become part of a community in which one population’s products may be used by another [72]. Such exchanges can distribute related metabolic functions among different organisms and contribute to the functional redundancy observed in complex microbial communities [14,149,150,151].

Clinical studies of probiotics and synbiotic formulations provide one line of evidence. Randomized, placebo-controlled trials have reported changes in microbial composition, metabolic pathways, and host-associated measurements under the conditions tested [60,152], alongside other multi-species synbiotic trials conducted in comparable metabolic populations [153,154]. These studies are presented here as illustrative examples of biological activity, but they do not establish mechanistic succession. Other publications cited in this section [124,135,151,155] provide indirect or contextual evidence, such as ecological mechanisms of resilience or general observations from microbiome research, rather than a direct evaluation of the formulations themselves. Taken together, these heterogeneous findings support the plausibility of individual components of the framework while underscoring that causal continuity across stages remains hypothetical and requires targeted, longitudinal validation.

What remains unproven is the proposed connection among these observations. Survival of a spore does not demonstrate that its vegetative activity remodeled an intestinal niche [156]. A change in the abundance of another organism does not show that the pioneer facilitated it. Likewise, an improvement in HOMA-IR or circulating LPS cannot be assigned to microbial succession unless the intervening events have been followed. The evidence presently available supports the plausibility of the individual stages, but not the entire sequence as a demonstrated mechanism.

This problem extends well beyond the studies considered here. Most clinical trials ask whether an intervention produces an effect; they are not designed to follow the ecological events that lead to it [99,157,158]. Samples are often collected at the beginning and end of treatment, and much of what happens in between is missed. Reliance on fecal material further limits the data because it provides little information about the small intestine, proximal colon, or mucosa-associated organisms. This limitation applies less directly to studies relying on blood-based endpoints, such as HOMA-IR and circulating lipopolysaccharide, rather than fecal community profiling. However, systemic markers likewise cannot localize events occurring in the mucosa or small intestine and should not be interpreted as direct evidence of community-level ecological remodeling. Failure to detect a proposed intermediate event may therefore reflect the timing or location of sample collection rather than its absence.

For this reason, judge the framework by the experiments it makes possible. If the predicted order of events is not observed, or if removal of an early stage has no effect on what follows, the model will require revision. Clinical benefit could still occur, but another mechanism would have to explain it. The model is not meant to accommodate every outcome. It identifies observations that would support or contradict the proposed ecological process.

### 5.3. Implications for the Design of Microbiome Therapeutics

If the model is supported, formulation design would begin with the ecological problem to be addressed. An intestine characterized by increased oxygen availability and expansion of facultative anaerobes presents a different problem from one depleted of fermentative organisms after antibiotic treatment [102,159]. Both conditions may be described as dysbiosis, but they are unlikely to respond in the same way to an identical microbial formulation.

This approach would require investigators to define each component’s intended function. Some organisms may be included because they survive transit and alter the local environment. Others may supply enzymes that release substrates from otherwise inaccessible dietary compounds. Still others may use the resulting metabolites or restore functions that are poorly represented in the resident community. Prebiotic ingredients would be selected according to the organisms expected to use them and the products expected to result, rather than added as general stimulants of microbial growth.

The order and timing of administration may prove as important as composition [160]. If secondary organisms depend on conditions created by pioneers, simultaneous delivery may not always be optimal. Conversely, separating the components by too long an interval may allow the initial environmental change to disappear before the secondary community becomes active. These possibilities can be examined directly by varying the sequence and timing of treatment.

Dose will also require further consideration. A larger number of viable organisms does not necessarily ensure a better response. For an organism serving as a pioneer, the important consideration may be whether it remains active long enough to produce the required environmental change [103]. Increasing the dose beyond that point could add little value and might alter acid production, substrate competition, or inhibition of other organisms in unintended ways. The appropriate dose may therefore depend as much on the organism’s activity and persistence as on the number administered.

An ecological approach may also improve the interpretation of nonresponse. Failure of an intervention does not necessarily mean that every component lacks biological activity. The process may have stopped at a particular stage. The pioneer may not have germinated, the environmental change may have been too small, or the recipient may have lacked the microbial partners needed to continue the sequence. Identifying where the process stopped would be more informative than classifying the entire intervention as effective or ineffective.

### 5.4. The Importance of the Baseline Microbiome

Differences among individuals are commonly viewed as an obstacle to determining the average effect of an intervention. In this case, however, those differences may help determine the response itself. Treatment begins with the microbial community already present in the recipient, and its composition and activity affect what the administered organisms can do.

A depleted microbiome may offer little resistance to incoming organisms but may lack the partners needed to complete a metabolic pathway [28,161]. A diverse but disrupted community may retain those functions while resisting the introduced organisms [162]. In other individuals, the required organisms may already be present, and treatment may act mainly by increasing their activity [163]. Similar clinical responses could therefore arise from different taxonomic changes, while similar taxonomic changes could have different consequences depending on the functions already present [116,164].

This may explain why baseline taxonomic markers alone have had limited success in predicting response. The relevant question is not simply which organisms are present. It is whether the community contains the functions, substrates, and interactions needed for the proposed sequence to proceed. Prediction will probably require information about microbial activity and the intestinal environment in addition to community composition.

The longer-term objective is to determine where the response is most likely to stop in a given recipient. It may then be possible to modify the intervention by selecting a different pioneer, changing the organisms or substrates that follow it, or altering the timing of administration. There is not yet sufficient evidence to personalize treatment in this way. Nevertheless, the model suggests how such an approach might eventually be investigated.

### 5.5. Limitations of the Framework

Dividing the proposed process into five stages is useful for experimental purposes, but it is an obvious simplification of what occurs within the intestine. The stages will probably not begin and end at clearly defined points. Pioneer organisms may remain active as other populations respond, and the host may begin to react before the microbial community has reached a stable state. That response may, in turn, change the intestinal environment and influence the community that is still developing.

The term “directed succession” also warrants careful consideration. Direction does not imply complete control or guarantee a beneficial outcome. At most, an intervention may increase the likelihood that the community will follow one trajectory rather than another. The resident microbiome and the host remain active participants and may oppose or redirect the intended process.

The evidence used to develop the framework did not come from a single type of study or comparable experimental conditions. Genomic analysis can show what an organism may be capable of doing, but it cannot show that the same activity occurs after ingestion. Laboratory models allow closer examination of intestinal conditions, although they reproduce only part of the host environment. Studies in animals and humans provide additional evidence, but each introduces different limitations. In particular, most clinical trials were not sampled often enough to determine whether the events proposed here occurred in sequence. The framework therefore brings together observations made at different levels of investigation, with the uncertainty that such a synthesis necessarily carries.

Another limitation is the transient nature of many reported microbiome changes. Several clinical trials of probiotics have failed to demonstrate lasting ecological remodeling, with observed shifts in microbial composition or metabolic activity disappearing after treatment withdrawal [136]. This underscores that persistence of altered functions cannot be assumed. The framework therefore requires longitudinal validation to distinguish temporary pharmacological or nutritional effects from durable ecological succession. Even if clinical benefit occurs, it may reflect short-term modulation rather than sustained ecological engineering.

The strains and formulations used to illustrate the framework do not independently prove the model. Evidence relevant to its individual stages also appears in studies of probiotic engraftment, defined bacterial consortia, microbiome recovery after antibiotics, and fecal microbiota transfer. Human studies show that establishing an administered strain depends on ecological opportunities within the recipient microbiome, and that recovery of probiotic organisms in feces does not necessarily reflect their activity at the mucosal surface [165,166]. Studies of bacterial consortium VE303 and SER-109 provide additional examples in which defined or spore-based microbial preparations influenced colonization, community recovery, microbial metabolites, and clinical outcomes [106,107,108]. Other experimental work has established plausible mechanisms involving epithelial oxygenation, metabolic cross-feeding, and treatment response dependence on the baseline microbiome [115,128,167,168]. None of these studies demonstrates the complete five-stage sequence, but together they broaden the evidence beyond the formulations that initially informed this framework.

### 5.6. Broader Significance

Although particular strains and formulations have been used to illustrate the model, it may apply to other organisms. A different microorganism could perform the pioneer role if it survives administration and becomes active where an environmental change is needed. The organisms that respond later need not be fixed either. Their importance lies in whether they can use, extend, or complement the activities initiated during the earlier stages.

The framework’s broader contribution is therefore not a particular combination of organisms. It is a way of deciding why the organisms should be combined and what must occur after they are administered. This changes the central question from whether an individual strain possesses a desirable property to whether a sequence of microbial activities can move a community toward a more persistent and useful functional state.

Whether this is possible remains an open question. The model provides a way to investigate this by tracking the activity of the administered organisms, the resident community’s response, and the effects observed in the host. If these events occur in the proposed order, future formulations could be selected based on the ecological functions their components perform together, rather than primarily on characteristics measured for one strain at a time. Safety is central to any attempt at ecological engineering [169], a concern that becomes more important, not less, as ecological engineering moves from traditional strain-survival and tolerance assessments toward deliberate and persistent ecosystem manipulation. The following section addresses these safety considerations in detail.

The proposed framework does not assume a single universal target state. Instead, it defines therapeutic success in terms of ecological functions appropriate to the disturbance being addressed. In metabolic disease, this may involve increased butyrate production and improved glycemic regulation; in post-antibiotic communities, recovery of fermentative partners and reduction of facultative anaerobes; in recurrent *C. difficile* infection, enhanced colonization resistance and stabilization of protective functions. In healthy adults, the target may be maintenance of resilience rather than change. Thus, “restoration” is reframed as achieving measurable ecological functions—functional redundancy, cross-feeding, barrier support, and host metabolic balance—rather than a vague return to a prior state. This clarification strengthens the framework by linking ecological engineering to disease-specific objectives and experimentally testable outcomes.

Table 4 explicitly summarizes these disease-specific targets, replacing the undefined concept of a single “restoration” endpoint with a defined ecological target for each clinical context discussed in this review.

### 5.7. Safety Considerations for Ecological Engineering

Extending microbiome therapeutics from transient supplementation toward deliberate, persistent ecosystem engineering substantially raises the safety bar. For spore-forming organisms and defined consortia intended to establish or persist within the intestinal community, evaluation must begin at the strain level: whole-genome screening for virulence factors, toxin-encoding genes, and other strain-specific pathogenic determinants, rather than relying on species-level safety history alone. A related concern is the presence of transferable antimicrobial-resistance determinants. Genes conferring resistance are common among gut commensals and can be mobilized by horizontal gene transfer through plasmids, transposons, and other mobile genetic elements; an organism engineered to persist and interact closely with the resident community offers correspondingly more opportunity for such transfer. Genomic and functional screening for mobilizable resistance genes should therefore be a prerequisite, not a supplement, for any consortium intended for sustained ecological engagement, as already performed for the spore-forming strains [170,171,172].

Beyond genomic risk, the metabolic activity of an engineered community introduces its own hazards. Establishing organisms with defined biosynthetic or catabolic capacities can generate metabolites at concentrations or in contexts not observed with transient supplementation, and administered or stimulated taxa may participate directly in bile-acid metabolism, where altered deconjugation and secondary bile-acid production have been linked to intestinal inflammation and carcinogenic risk. Translocation is a further concern, particularly in vulnerable hosts—critically ill, immunocompromised, or otherwise barrier-compromised individuals—in whom organisms that behave innocuously in an intact gut may gain systemic access; this concern applies with at least equal force to interventions designed to engage the mucosal environment more persistently.

Finally, and perhaps most distinctively for this framework, ecological displacement raises a possibility distinct from conventional adverse events: a successfully engineered, stable community configuration is not automatically desirable. Persistence and stability are properties of a state, not evidence of its benefit; competitive exclusion of an administered organism could equally remove a beneficial resident population, and a new configuration could stabilize around a functionally undesirable or even pathogenic-permissive state as readily as around a beneficial one. This becomes more important, not less, as interventions move from transient supplementation toward deliberate and persistent ecosystem manipulation, because a stable but undesirable configuration may be considerably harder to reverse than a transient one. Safety evaluation within the proposed framework therefore cannot be limited to strain-level screening before administration; it must include ecological monitoring afterward—tracking functional as well as compositional endpoints at each stage—so that persistence is assessed against whether the resulting configuration is beneficial, not merely whether it is stable.

## 6. Conclusions and Future Directions

The gut microbiome is often treated as a collection of organisms whose individual properties can be selected and combined to produce a therapeutic effect. This approach has yielded useful probiotics and greatly advanced our understanding of individual strains. However, it does not fully account for the community into which those organisms are introduced. The intestinal microbiome is already established, and its response will depend on the organisms and functions present when treatment begins.

The framework proposed in this review considers whether selected microorganisms can be used in a defined ecological sequence. Spore-forming organisms may serve as transient pioneers if they survive administration, germinate, and alter intestinal conditions. Those changes could then affect resident populations or organisms supplied as part of a synbiotic consortium. If the resulting functions become distributed among several community members, some may persist after the administered organisms are no longer detected. A host response would represent the final stage of this process.

Evidence supports the biological plausibility of the individual stages. Studies have demonstrated spore survival and germination, metabolic exchange among intestinal microorganisms, environmental control of community composition, recipient-dependent engraftment, and changes in host measurements following microbiome interventions. What has not been demonstrated is that these events occur in the order proposed here or that each depends on the one preceding it. The five-stage framework should therefore not be regarded as an established mechanism of probiotic or synbiotic action.

The next experiments should follow the process as it occurs. This will require sampling at intervals that can detect early microbial activity, along with measurements of the intestinal environment, microbial gene expression, metabolic products, and host response. Studies comparing the individual components of an intervention with the complete formulation will be particularly important. Changing the order of administration or removing a proposed pioneer function would directly test whether later events depend on earlier ones. Follow-up after treatment has ended will also be needed to determine whether an altered function persists or disappears with the administered organisms.

The value of ecological engineering will ultimately depend on whether it improves the predictability of microbiome intervention. The framework may prove incomplete, and different starting communities may follow different trajectories. Even so, determining where the proposed sequence succeeds or fails would provide information endpoint comparisons alone cannot provide. The central question is no longer which strain to administer. It is whether the activities of several organisms can be arranged so that an initial ecological change permits other microbial functions to develop and, under appropriate conditions, to be maintained.

## Figures and Tables

**Table 1:** Comparison of current microbiome-directed strategies and the ecological considerations relevant to their design.

Strategy	Primary Objective	Ecological Limitation	Implication for Ecological Design
Conventional probiotics	Delivery of one or more selected strains [61,62,63]	Activity and persistence depend on the resident microbiome and the availability of a suitable niche [36,38,39,45]	Benefit may arise from transient activity; permanent engraftment should not be assumed or required
Synbiotics	Delivery of microorganisms together with substrates intended to support microbial activity [72,73,74]	Outcomes depend on whether the substrate is used by the intended organisms and whether their functions complement those already present [72,73,74]	Organisms and substrates should be selected according to the metabolic relationships expected after administration [60,73,94]
Live biotherapeutic products	Administration of defined live organisms for the prevention, treatment, or management of disease [77,78]	Product characterization does not by itself predict behavior within a complex recipient community [38,39,61]	Development should include measurements of ecological interactions and community-level function [77,79,85,86]
Fecal microbiota transplantation	Transfer of a complex donor-derived microbial community [86,87]	Composition is incompletely defined, and the behavior of the transferred community depends on both donor and recipient factors [88,104,105]	Defined consortia may provide greater experimental control, although they reproduce only part of the complexity of a complete community [89,106,107,108]

**Table 2:** Proposed ecological processes, biochemical mechanisms, and anticipated system-level outcomes during facilitated community assembly.

Ecological Process	Biochemical Mechanism	Emergent System Outcome
Metabolic Cross-Feeding	Pioneer amylases and glycoside hydrolases break down complex prebiotics (inulin) into short oligosaccharides [129,130].	Secondary obligate anaerobes utilize these intermediates to fuel high-rate butyrogenesis Evidence basis: inferred from genomic/enzymatic characterization of this exact strain [93,94,111] and general cross-feeding physiology; the downstream butyrogenic effect has not been directly measured for this formulation and remains hypothetical.
Glycemic Shunting	Specific strains endogenously convert dietary glucose and fructose into D-mannitol [125].	Reduction of luminal monosaccharide availability for pathobionts; provision of alternative fermentable substrates [127,131]. Evidence basis: the mannitol-conversion mechanism is demonstrated in a different, non-probiotic organism (recombinant *E. coli*) [125], not in the strains discussed here; the downstream competitive-exclusion outcome is inferred from unrelated gut-ecology studies [112,127] and remains hypothetical for this system.
Metabiotic Signaling	Enzymatic lysate of *Lactobacillus delbrueckii* delivers non-viable microbial constituents with preserved bioactive components [71].	Enhanced functional delivery independent of in vivo replication; increased robustness across heterogeneous baseline microbiomes. Evidence basis: hypothetical; general microbiome-resilience literature is cited [71], but strain-specific data on enzymatic-lysate signaling for this formulation are not yet available.
Niche Expansion & Exclusion	Potential co-localization of spore-forming probiotics and synbiotic strains within the mucus layer, suggested by the presence of adhesin (*spa*CBA, *eno*) [111,132].	Hypothetical niche exclusion and pathobiont suppression, requiring direct validation through strain-resolved gene expression, mucosal localization assays, and functional activity studies. Evidence basis: inferred from genomic annotation of this exact strain (adhesin genes) [93,94]; explicitly hypothetical and not yet functionally validated, as noted in this row.
Redox Stabilization	Butyrate-producing taxa fuel colonocyte β-oxidation, reinforcing mucosal hypoxia and suppressing facultative anaerobe expansion [133,134]	Establishment of a strict anaerobic filter that stabilizes the Lachnospiraceae:Enterobacteriaceae (LE) ratio [135]. Evidence basis: mechanism demonstrated in unrelated host-microbiome systems, primarily animal models [133,134]; not yet demonstrated for the strains discussed in this review.

**Table 3:** Experimental requirements for evaluating the five-stage ecological engineering framework.

Hypothesis/Ecological Stage	Primary Biological Level	Key Experimental Approaches	Critical Validation Criterion
H1/Stage 1 & 2: Pioneer Germination as Allogenic Ecological Filter	Microbial niche remodeling [94,102,112,113,114]	Humanized continuous bioreactor models: Simulator of the Human Intestinal Microbial Ecosystem (SHIME) [65]; real-time microsensor measurements of dissolved oxygen (O_2_) and pH [102,113]; metatranscriptomics (for facultative anaerobes) [114,115]. SHIME is a validated in vitro gastrointestinal simulator, not a genuinely humanized host model; it does not reproduce epithelial oxygen consumption, immune responses, mucus turnover, absorption, or host feedback simultaneously. Mechanistic completion of Stages 1–3 in this system should therefore be interpreted as evidence of biological plausibility, not as demonstration that the same succession occurs in vivo, and will require complementary human validation.	Suggestion of causal temporal sequence: pioneer germination → environmental remodeling → pathobiont suppression Requires complementary validation in human studies to confirm succession in vivo
H2/Stage 3: Synergistic Metabolic Outputs from Cross-Feeding	Microbial metabolic cooperation	Randomized four-arm design (placebo, spore-formers, synbiotic, combined); GC-MS metabolomics; stable isotope probing (SIP) with ^13^C-labeled substrates.	Demonstration of synergistic (non-additive) metabolic outputs Short-Chain Fatty Acids (SCFAs), mannitol) in the combined arm.
H3/Stage 4: Directed Succession and Functional Redundancy	Ecosystem resilience	Long-term clinical monitoring with extended washout; longitudinal shotgun metagenomics; ecological network analysis (nestedness, modularity).	Persistence of functional redundancy and network resilience after treatment cessation.
H4/Stage 5: Ecological Remodeling and Host Adaptation	Host metabolic response	Correlation analysis linking multi-omic microbiome profiles to systemic biomarkers (LPS, HOMA-IR); human intestinal organoids exposed to metabolic supernatants; Transepithelial Electrical Resistance (TEER) measurement.	Demonstration of causal chain: ecological remodeling → epithelial reinforcement → resolution of endotoxemia → improved insulin sensitivity.

**Table 4:** Disease-specific ecological targets proposed by the framework.

Clinical/Ecological Context	Primary Ecological Target(s)	Rationale
Healthy adults	Maintenance of resilience (return to baseline after perturbation)	No disturbance to correct; the goal is preserving existing functional capacity rather than producing change.
Metabolic disease (prediabetes, type 2 diabetes)	Increased butyrate production; improved glycemic regulation	Short-chain fatty acid signaling and barrier support are mechanistically linked to insulin sensitivity and glycemic control.
Post-antibiotic communities	Recovery of fermentative partners; reduction of facultative anaerobes	Antibiotic disturbance selects for facultative anaerobes and depletes obligate fermenters; recovery of the latter marks functional restitution.
Recurrent Clostridioides difficile infection	Enhanced colonization resistance; stabilization of protective functions	Loss of colonization resistance is the proximate mechanism of recurrence; its restoration is the direct therapeutic target.

## Data Availability

No new data were created or analyzed in this study. Data sharing is not applicable to this article.
